# Association of variations in platinum resistance-related genes and prognosis in lung cancer patients

**DOI:** 10.7150/jca.44410

**Published:** 2020-04-27

**Authors:** Yuan-Kang Zhou, Xiang-Ping Li, Ji-Ye Yin, Ting Zou, Zhan Wang, Ying Wang, Lei Cao, Juan Chen, Zhao-Qian Liu

**Affiliations:** 1 Department of Clinical Pharmacology and National Clinical Research Center for Geriatric Disorder, Xiangya Hospital, Central South University, Changsha 410008, China; 2 Institute of Clinical Pharmacology, Central South University, Hunan Key Laboratory of Pharmacogenetics, Changsha 410078, P. R. China; 3 Department of Pharmacy, Xiangya Hospital, Central South University, Changsha 410008, China; 4 Department of National Institution of Drug Clinical Trial, Xiangya Hospital, Central South University, Changsha 410008, China; 5 Department of Lung Cancer and Gastroenterology, Hunan Cancer Hospital, Affiliated Tumor Hospital of Xiangya Medical School of Central South University, Changsha, 410013, China; 6 Department of the Central Laboratory, Hunan Cancer Hospital, Affiliated Tumor Hospital of Xiangya Medical School of Central South University, Changsha, 410013, China

**Keywords:** lung cancer, platinum-based chemotherapy, prognosis, polymorphisms, *REV3L*, * HMGB1*

## Abstract

**Purpose**: We aimed to investigate the association of single-nucleotide polymorphisms (SNPs) in *HMGB1*, *REV3L*, and *NFE2L2* with prognosis in lung cancer patients with platinum-based chemotherapy.

**Methods**: We have recruited 348 lung cancer patients treated with platinum. Log-rank test and Cox regression analysis were used to assess overall survival (OS) and progression-free survival (PFS) among SNP genotypes.

**Results**: The results revealed that patients carrying TC or CC genotype in *REV3L* rs462779 (HR=0.67, 95% CI=0.51-0.90, *P*=0.007) and AG or GG genotype in *HMGB1* rs1045411 (HR=0.61, 95% CI=0.38-0.99, *P*=0.046) had a better overall survival. Additionally, carrying TC or TT genotype in rs462779 had a lower risk (OR=0.38, 95% CI=0.17-0.89, *P*=0.025) of lymph node metastasis, carrying AG or AA genotype in rs1045411 was significantly related to early T stage (OR=0.47, 95% CI=0.29-0.76, *P*=0.002). In stratified analysis, patients with TC or CC genotype in rs462779 were significantly associated with overall survival in male patients, never-smokers, patients with younger age (≤56), no family history of cancer, adenocarcinoma, advanced stage (stage III or IV), or ECOG PS 0-1. While patients with AG or GG genotype in rs1045411 were significantly associated with overall survival in patients with advanced stage (stage III or IV) or ECOG PS 0-1.

**Conclusion**: Our results indicate that the TC or CC genotype in rs462779 and AG or GG genotype in rs1045411 are contributed to better overall survival. The *REV3L* rs462779 and *HMGB1* rs1045411 may serve as prognosis markers in lung cancer patients with platinum-based chemotherapy.

## Introduction

According to recent studies, lung cancer is still the leading cause of cancer mortality both in males and females, which accounts for 18.4% of total cancer death 1. A majority of lung cancer patients, approximately 85%, are diagnosed with non-small cell lung cancer (NSCLC) 2. To the best of our knowledge, tobacco smoking is the main risk factor for lung cancer, and other factors are environmental- and occupational-related. Though the incidence rate has slightly decreased in recent years, the 5-year survival of lung cancer is merely 19% 3. Most of the patients have been diagnosed with an advanced stage, the prognosis for patients with lung cancer is still depressing. Obviously, the prognosis of lung cancer patients with different stage varies greatly, it is necessary to improve survival by finding biomarkers for prognosis prediction. In addition to traditional prognostic factors including tumor microenvironment state and clinical stage, diverse outcomes of cancer patients may also attribute to genetic alterations 4.

In spite of other treatments, like surgery, targeted therapy, radiation, and immunity therapy, platinum-based chemotherapy is still the first-line treatment for lung cancer patients 5. Platinum-based chemotherapy drugs mainly function by forming DNA adducts intrastrand and interstrand, cells recognize the adducts as DNA damage then activate DNA repair mechanisms, like nucleotide excision repair, mismatch repair, base-excision repair, non-homologous end-joining, and homologous recombination 6, 7. The initial response of platinum in most lung cancer patients is generally good, but intrinsic or acquired resistance would influence the sensitivity of platinum. The underlying mechanisms are described as following: increased damage, decreased accumulation, detoxification, epigenetic changes, decreased apoptosis, membrane trafficking changes, and genetic variants 8-10.

Platinum resistance-related genes affect the efficacy of platinum-based chemotherapy, which may act on the prognosis of patients. There are many genes associated with platinum resistance, according to database searching and literature references, we chose *HMGB1*, *REV3L*, and *NFE2L2* to study the association between genetic variations and clinical outcome. *HMGB1* (high mobility group box 1) is situated in the nucleus to maintain nuclear homeostasis, which plays an indispensable role in many diseases and cellular processes, like tumor, inflammation, cell differentiation, and migration 11. *HMGB1* directly binds to lesions of DNA then pursuits to DNA repair and tends to relate with platinum toxicity and liver damage 12, 13. There are many reports about variants of *HMGB1*. The rs1045411 is related to the overall survival of gastric cancer and the risk of developing lung cancer, hepatocellular carcinoma, and lymph node metastasis of breast cancer 14-17. The rs1412125 and rs2249825 appear to be significantly related to the risk of lung cancer and platinum response and seem to relate with the progression of breast cancer 15, 17-19. *REV3L* (the zeta catalytic subunit of DNA polymerase) is the most important DNA polymerase, it plays a role in maintaining genome stability in the advent of DNA damage and cell proliferation after damage, also may inhibit tumorigenesis 20, 21. Inhibition of *REV3L* sensitizes lung cancer and gliomas to cisplatin chemotherapy 22, 23. The rs462779 and rs465646 in *REV3L* are associated with overall survival in platinum-treated malignant mesothelioma, rs462779 is also correlated with event-free survival and risk of colorectal cancer 24, 25. Transcription factor *NFE2L2* (nuclear factor, erythroid 2 like 2), also named *NRF2*, functions as an oncogene or tumor suppressor, it regulates the cellular antioxidant response and promotes cancer chemotherapy resistance, metastasis, and progression 26-28. The rs6706649 and rs6721961 in *NFE2L2* may influence breast cancer prognosis, rs6721961 and rs35652124 are associated with susceptibility to colorectal cancer 29, 30.

In this study, we investigate the associations of *HMGB1* (rs1045411, rs1412125, and rs2249825), *REV3L* (rs462779 and rs465646) and *NFE2L2* (rs6706649, rs6721961, and rs35652124) with platinum-based chemotherapy prognosis in lung cancer patients.

## Materials and methods

### Subjects

351 lung cancer patients were recruited in the study between November 2011 and May 2013 in Xiangya Hospital of Central South University and Hunan Provincial Tumour Hospital in Changsha, Hunan Province, and clinical data were collected in the same place. No patients had received surgery, targeted therapy, radiotherapy, and/or biological therapy before first-line chemotherapy. In demographic characteristics study and outcome analysis, we excluded 3 patients who had incomplete clinical information. In tumor progression analysis, we excluded 46 patients who had incomplete TNM stage information. All subjects were Han people of Chinese and successfully genotyped after donated 5 mL blood, all patients were treated with platinum-based therapy, and each had signed informed consent. A standard follow-up was carried out to collect characteristic data. Clinical data including age, gender, smoking history, family history of cancer, histology classification, TNM stage, and Eastern Cooperative Oncology Group Performance Status. The last follow-up date was in July 2019.

### SNPs selection and genotyping

*HMGB1* SNPs (rs1045411, rs1412125, and rs2249825), *REV3L* SNPs (rs462779 and rs465646), and *NFE2L2* SNPs (rs6706649, rs6721961, and rs35652124) were selected for genotyping in the Han Chinese patients. They were chosen according to the following criteria: (1) based on our previous research, *HMGB1* SNPs were related with lung cancer platinum-based chemotherapy response 18, (2) association with the outcome of cancers and involvement in other cancer types in other research, (3) functional relevance of gene transcription or protein expression.

EDTA tube was used to hold 5 mL of venous blood donated from each patient. DNeasy Blood & Tissue Kit (Qiagen, Shanghai, China) or Genomic DNA Purification Kit (Promega, Madison, WI, USA) was used to extracting genomic DNA based on the instructions. Sequenom Mass Array Genotype Platform (Sequenom, San Diego, CA, USA) was used to genotype SNPs of each gene. Primers were designed using Primer-Premier 6 software (Premier Biosoft Interpairs, Palo Alto, CA). The Sequenom Mass Array Genotype Platform was used to design polymerase chain reaction (PCR), the shrimp alkaline phosphatase enzyme (SAP) reaction and associated extension reactions. The PCR system was heating for 15 minutes at 94℃, thermocycling for 45 times (94℃ for 20 seconds, 56℃ for 30 seconds, then 72℃ for 1 minute) and extension at 72℃ for 3 minutes. The SAP reaction was performed at 37℃ for 40 minutes then 85℃ for 5 minutes. The extension reaction was conducted at 94℃ for 30 seconds, 94℃ for 5 seconds, 40 cycles for 5 seconds at 52℃, five cycles for 5 seconds at 80℃, finally 72℃ for 3 minutes. The resin was used to purify the reaction product, and the Mass Array system (Sequenom) was used to resolve the data.

### Statistical analysis

The SPSS version 25.0 (SPSS Inc., Chicago, IL, USA) and GraphPad Prism (version 8, GraphPad Software Inc., San Diego, CA) were used for statistical analysis. Three genetic models (additive, dominant, and recessive model) were applied to evaluate the association between SNPs and prognosis of lung cancer patients. Overall survival (OS) and progression-free survival (PFS) were analyzed as endpoints and were defined as the time from diagnosis to death for any reason, the time from diagnosis to the first time when patients progressed, respectively. The association between clinical or SNPs data and OS or PFS was assessed using Cox proportional hazards regression analysis, computed as HRs with corresponding 95% CIs. Binary logistic regression was used to test the difference in disease progression between groups. The log-rank test was used to examine the difference in overall survival or progression-free survival between groups. Kaplan-Meier plot was used to visualize the results. All the *P*-values were two-sided, *P*<0.05 were supposed to be significant.

## Results

### Demographic characteristics of patients and prognosis analysis

A total of 348 subjects were included in this investigation, all had received platinum-based chemotherapy. As shown in Table 1, the median age was 56 years (a range of 21 to 75 years). Among them, 78 (22.4%) were females, 270 (77.6%) were males. There were 205 (58.9%) patients who had ever smoked, and the rest 143 (41.1%) patients never smoked. Most of the patients (95.4%) did not have a family history of cancer. For histology, 179 (51.4%) patients were diagnosed with adenocarcinoma, 142 (40.8%) with squamous cell carcinoma, the remainings with other types. 338 (97.1%) of the patients were in an advanced stage (stage III or IV). Likewise, most patients (94.0%) were with Eastern Cooperative Oncology Group Performance Status (ECOG PS) 0 or 1. In the univariate Cox regression analysis, histology was significantly associated with progression-free survival, the risk of progression in patients with squamous cell carcinoma (HR=0.69, 95% CI=0.54-0.89, *P*=0.004) was lower than that in patients with adenocarcinoma** (Table 1)**.

### Relationship between the eight SNPs and clinical outcome of platinum-based chemotherapy

Log-rank test and multivariate Cox regression analysis with adjustment for age, gender, and histology were conducted to analyze the association between the eight SNPs and OS or PFS. The rs462779 in *REV3L* was significantly correlated with overall survival of lung cancer patients in the additive (*P* for log-rank=0.018) and recessive (*P* for log-rank=0.005) models (**Table 2**). Patients carrying TC or CC genotype in rs462779 showed a markedly lower death risk (HR=0.67, 95% CI=0.51-0.90, *P*=0.007) when compared with TT genotype in the recessive model** (Table 2)**. There was also a trend difference in progression risk that patients with TC or CC genotype had better progression-free survival (*P* for log-rank=0.034), while the *P*-value for Cox regression (HR=0.77, 95% CI=0.59-1.02, *P*=0.073) was not significant (**Table 2**). Additionally, patients carrying AG or GG genotype in rs1045411 were significantly related to better overall survival (HR=0.61, 95% CI=0.38-0.99, *P*=0.046) **(Table 2)**. The other SNPs were not significantly associated with either OS or PFS.

### Association between the eight SNPs and lung cancer progression

To investigate whether the eight single nucleotide polymorphisms were related to tumor progression, binary logistic regression was used to analyze the association. As shown in Table 3, patients carrying *REV3L* rs462779 TC or TT genotypes had a lower hazard (OR=0.38, 95% CI=0.17-0.89, *P*=0.025) of lymph node metastasis in the dominant model when compared with patients carrying CC genotype. The rs1045411 and rs2249825 in *HMGB1* gene were significantly associated with T stage. Patients with AG or AA genotype in rs1045411 were significantly related to early T stage (T1 or T2) when compared with GG genotype (OR=0.47, 95% CI=0.29-0.76, *P*=0.002), and patients with GC or GG genotype in rs2249825 were also associated with early T stage when compared with CC genotype (OR=0.56, 95% CI=0.33-0.94, *P*=0.028) **(Table 3)**.

### Subgroup analysis of association between *REV3L*/*HMGB1* polymorphisms and prognosis

**Figure 1:**
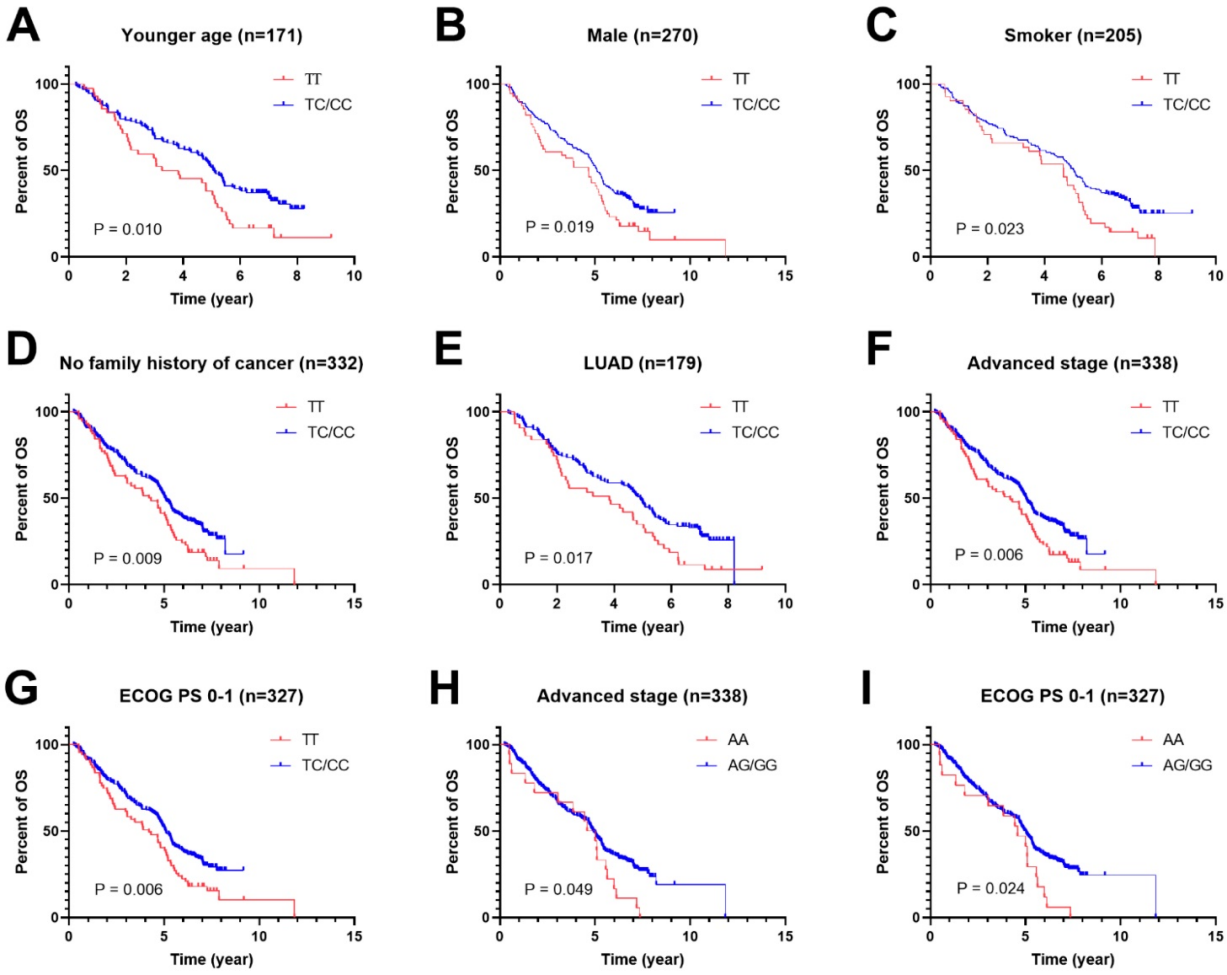
** Kaplan-Meier plots of stratified analysis of lung cancer patients with rs462779 (A-G) or rs1045411 (H-I) in recessive model. A,** Overall survival of patients with age ≤56 years old. **B,** Overall survival of male lung cancer patients. **C,** Overall survival of lung cancer patients who had never smoked. **D,** Overall survival of lung cancer patients without a family history of cancer. **E,** Overall survival of lung cancer patients with adenocarcinoma. **F,** Overall survival of lung cancer patients with an advanced tumor (stage III or IV). **G,** Overall survival of lung cancer patients with Eastern Cooperative Oncology Group Performance Status 0 or 1. **H,** Overall survival of lung cancer patients with an advanced tumor (stage III or IV). **I,** Overall survival of lung cancer patients with Eastern Cooperative Oncology Group Performance Status 0 or 1.

To further study the associations of rs462779 and rs1045411 with the prognosis of platinum-based chemotherapy, multivariate Cox proportional hazard analysis was performed in the recessive model stratified by clinical characteristics. In the patients with younger age (≤56 years old), we observed that the TC or CC genotype in rs462779 had significantly lower death risk (HR=0.61, 95% CI=0.42-0.91, *P*=0.015) when compared with TT genotype. For male patients, the TC or CC genotype also had significantly lower death risk (HR=0.69, 95% CI=0.50-0.96, *P*=0.027). For patients who had ever smoked, carrying TC or CC genotype had significantly lower death risk (HR=0.65, 95% CI=0.45-0.95, *P*=0.026). For patients without a family history of cancer, the HR for death of carrying TC or CC genotype was 0.68 (95% CI=0.51-0.92, *P*=0.011). In adenocarcinoma subgroup, the hazard ratio for death was 0.65 (95% CI=0.45-0.94, *P*=0.023). Those with advanced stage (stage III or IV) carrying TC or CC genotype had lower death risk (HR=0.68, 95% CI=0.51-0.91, *P*=0.009). For those who had Eastern Cooperative Oncology Group Performance Status 0 or 1 score, the HR for death of carrying TC or CC genotype was 0.67 (95% CI=0.49-0.90, *P*=0.008)** (Supplementary Table 1, Figure 1A-1G)**.

For *HMGB1* rs1045411, patients with advanced stage (stage III or IV) carrying AG or GG genotype had lower death risk (HR=0.62, 95% CI=0.38-1.00, *P*=0.050) when compared with AA genotype. For patients with ECOG PS 0 or 1 score, the hazard ratio for death of carrying AG or GG genotype was 0.57 (95% CI=0.35-0.93, *P*=0.025)** (Supplementary Table 2, Figure 1H and 1I)**.

## Discussion

Platinum remains the commonly used drug for lung cancer patients, it is generally used in combination with other antitumor drugs, but the appearance of drug resistance leads to unsatisfied efficacy. Stable DNA adducts formed by cisplatin lead to DNA damage then mainly pursuit to nucleotide excision repair, finally result in cell apoptosis 6, 7. Enhanced DNA repair contributes to platinum resistance, which compromises the chemotherapy efficacy and therefore influences clinical outcome 10.

Polymorphism is one of the factors that may affect prognosis, and variants of genes of drug transporters, metabolic enzymes, DNA repair system, apoptosis pathway, and folate metabolism pathway are the most studied biomarkers for predicting platinum-based chemotherapy response, while more meaningful markers still need to be discovered 10.

In the present study, we focused on the genetic alterations of *HMGB1*, *REV3L*, and *NFE2L2*, a total of eight SNPs, to study the prognostic effect of platinum-based chemotherapy. Among them, *REV3L* rs462779 and *HMGB1* rs1045411 were significantly associated with overall survival; none of them was significantly associated with progression-free survival. In male patients, patients who never smoked, or patients with younger age (≤56), no family history of cancer, adenocarcinoma, advanced tumor (stage III or IV), or ECOG PS 0-1, patients carrying TC or CC genotype in rs462779 had better overall survival when compared with TT genotype. Patients carrying AG or GG genotype in rs1045411 had better overall survival in lung cancer patients with advanced stage (stage III or IV) or ECOG PS 0-1. In terms of tumor progression, carrying TC or TT genotype in *REV3L* rs462779 had a lower risk of lymph node metastasis when compared with CC genotype, while carrying AG or AA genotype in *HMGB1* rs1045411 or carrying GC or GG genotype in *HMGB1* rs2249825 were significantly related to early T stage (T1 or T2).

*REV3L* is an important DNA polymerase involved in DNA replication, repair, recombination, and has an increased mRNA expression in non-small cell lung cancer tissue, the C-terminal portion encompasses conserved DNA polymerase domain, and the N-terminal domain contains residues direct contacting to DNA 20, 31-33. Pol zeta plays a major extender role in translesion DNA synthesis (TLS) after DNA lesions due to its capability to extend mismatched or distorted primer templates of sorts, the lesions were directly bypassed by specialized DNA polymerases, such as DNA polymerases zeta 34, 35. Loss expression of *REV3L* increases the frequency of chromosome translocation and break, thus results in genomic instability, and it acts on as a tumor suppressor because of inhibition of spontaneous tumor formation 31, 35, 36. *REV3L* rs462779 is a nonsynonymous SNP (p.Thr1224Ile) that may influence protein function, *REV3L* rs465646 alters the microRNA binding site in the 3´-UTR, thus may affect *REV3L* expression 24.

Genetic variations in *REV3L* have been reported that it was associated with tumor risk or survival in multiple kinds of tumors. Patients carrying the TC or TT genotype in rs462779 had significantly increased colorectal cancer risk, and *REV3L* rs462779 and *RAD18* rs373572 seemed to have a strong cumulative relation with CRC risk, while carrying the TC or CC genotype in rs465646 had significantly decreased lung cancer risk 25, 37. Carrying the TC or CC genotype in rs462779 or carrying the AG or GG genotype in rs465646 was associated with good overall survival in platinum-treated malignant mesothelioma patients (n=139), the TC or CC genotype in rs462779 had poor overall survival in osteosarcoma patients (n=66), the TC or CC genotype in rs462779 had poor event-free survival in aggressive breast cancer patients (n=738) 24, 38, 39.

Furthermore, *REV3L* rs462779 showed a slight association with severe toxicity (thrombocytopenia) in NSCLC patients treated with platinum-based chemotherapy 40. Nonetheless, there is no report about the association between OS or PFS and polymorphisms in the platinum resistance-related *REV3L* gene. In our research, carrying TC or CC genotype in rs462779 had good overall survival in lung cancer patients (n=348), but no SNP was significantly associated with progression-free survival. It perhaps due to the differences in sample size and tumor type compared with previous studies. Besides, patients carrying TC or TT genotype had a lower risk of lymph node metastasis. However, the mechanisms of how rs462779 influences overall survival and lymph node metastasis still need further investigation.

*HMGB1* plays nuclear factor role or extracellular signaling molecule role during cell migration and tumor metastasis. Overexpression of *HMGB1* induced by chemotherapy or radiotherapy was associated with all hallmarks of cancer, which leads to tumor microenvironment disorder 12, 13, 41. Recent studies also show that *HMGB1* is involved in positive regulation and maintenance of ferroptosis in acute myeloid leukemia (AML) cells and autophagy in thyroid cancer cells, also prevents necroptosis in AML cells 42-44. On the aspect of cancer risk, rs1045411 was significantly associated with susceptibility to hepatocellular carcinoma, oral squamous cell carcinoma, lung cancer, breast cancer, and urothelial cell carcinoma 15-17, 45, 46. In terms of association of genetic variants of *HMGB1* and prognosis, previous reports showed that carrying AG or AA genotype of rs1045411 in *HMGB1* gene was significantly associated with good overall survival in gastric cancer patients 14. *HMGB1* rs1045411 is located in the 3'UTR, and was reported to decrease *HMGB1* expression through *hsa-miR-505-5p* 45, 47. *HMGB1* rs1045411 C/T heterozygous polymorphism was associated with a significantly lower ratio of developing EGFR mutation in the smoking population; it may be a protective factor in lung adenocarcinoma 47. In our study, carrying AG or GG genotype in rs1045411 was significantly associated with good overall survival. Moreover, rs1045411 and rs2249825 were significantly related to the early T stage.

There are also some limitations in our study. The recruitment hospitals are located in the same region, and the multi-central clinical studies may be needed to overcome results bias. It is a retrospective study with the sample size not large enough, therefore prospective or other analogous studies are warranted to validate our results. Thus we are enrolling other patients and independent validation will be done in our next studies. The underlying mechanism of rs462779 relating with cancer prognosis still needs further investigation.

In conclusion, our study suggested that *REV3L* rs462779 was significantly associated with overall survival and lymph node metastasis in lung cancer patients with platinum-based chemotherapy, and *HMGB1* rs1045411 was related to overall survival and T stage. Genotypes of *REV3L* rs462779 and *HMGB1* rs1045411 may be biomarkers for predicting platinum-based chemotherapy prognosis in lung cancer patients.

## Supplementary Material

Supplementary tables.

## Figures and Tables

**Table 1:** Distribution of characteristics in lung cancer patients and prognosis analysis (n=348)

Variables	Overall survival (OS)					Progression-free survival (PFS)				
	MST (mo)	Death/Total	P1	HR (95% CI)	P2	MST (mo)	Progression/Total	P1	HR (95% CI)	P2
Age (year)										
≤56	59.0	123/171	0.968	Ref.		33.2	139/171	0.078	Ref.	
>56	60.1	131/177		1.00 (0.78-1.27)	0.968	50.3	137/177		0.81 (0.64-1.03)	0.079
Gender										
Female	56.7	55/78	0.971	Ref.		35.5	61/78	0.890	Ref.	
Male	60.5	199/270		1.01 (0.75-1.36)	0.971	42.4	215/270		0.98 (0.74-1.30)	0.890
Smoking status										
Never smoker	60.1	101/143	0.401	Ref.		35.1	114/143	0.541	Ref.	
Ever smoker	59.0	153/205		1.11 (0.87-1.43)	0.401	46.1	162/205		0.93 (0.73-1.18)	0.541
Family history of cancer										
No	59.1	242/332	0.646	Ref.		39.1	263/332	0.808	Ref.	
Yes	52.9	12/16		1.15 (0.64-2.05)	0.646	38.6	13/16		1.07 (0.61-1.87)	0.808
Histology										
LUAD	56.1	137/179	0.172	Ref.		29.0	150/179	**0.014**	Ref.	
LUSC	63.1	98/142		0.78 (0.60-1.01)	0.062	56.5	104/142		0.69 (0.54-0.89)	**0.004**
Others^a^	60.5	19/27		0.92 (0.57-1.49)	0.740	31.3	22/27		0.94 (0.60-1.46)	0.767
TNM stage										
Ⅰ/Ⅱ	66.8	7/10	0.606	Ref.		54.3	8/10	0.729	Ref.	
III/IV	58.8	247/338		1.22 (0.57-2.58)	0.607	36.8	268/338		1.13 (0.56-2.29)	0.729
Eastern Cooperative Oncology Group Performance Status										
0-1	59.6	236/327	0.362	Ref.		41.4	258/327	0.516	Ref.	
>1	48.6	18/21		1.25 (0.77-2.02)	0.363	22.2	18/21		1.17 (0.73-1.89)	0.517

MST, median survival time; mo, month; HR, hazard ratio; CI, confidence interval; P1, *P*-value for log-rank test; P2, *P*-value for univariate Cox hazards regression analysis; Ref., reference; LUAD, lung adenocarcinoma; LUSC, lung squamous cell carcinoma. *P* < 0.05 are indicated in bold text. ^a^Other carcinomas include adenosquamous carcinoma, large cell carcinoma, bronchogenic lung cancer, and mucoepidermoid carcinoma.

**Table 2:** Association between single nucleotide polymorphisms (SNPs) and platinum-based chemotherapy prognosis (n=348)

Gene/SNP	Model	Genotype	Overall survival (OS)					Progression-free survival (PFS)				
			Death/total	MST (month)	P1	HR (95% CI)	P2	Progression/total	MST (month)	P1	HR (95% CI)	P2
HMGB1												
rs1045411	Additive	GG	161/221	59.1	0.127	Ref.		174/221	41.4	0.698	Ref.	
		AG	75/109	60.0		0.97 (0.74-1.28)	0.850	84/109	35.1		1.04 (0.80-1.35)	0.788
		AA	18/18	57.5		1.62 (0.99-2.65)	0.054	18/18	54.1		1.21 (0.74-1.97)	0.444
	Dominant	GG	161/221	59.1	0.796	Ref.		174/221	41.4	0.687	Ref.	
		AG/AA	93/127	60.0		1.06 (0.82-1.37)	0.677	102/127	35.8		1.06 (0.83-1.36)	0.621
	Recessive	AA	18/18	57.5	**0.045**	Ref.		18/18	54.1	0.404	Ref.	
		AG/GG	236/330	59.1		0.61 (0.38-0.99)	**0.046**	258/330	36.8		0.84 (0.52-1.35)	0.464
rs1412125	Additive	TT	133/182	58.1	0.998	Ref.		145/182	36.6	0.495	Ref.	
		CT	104/143	59.1		0.99 (0.76-1.28)	0.910	114/143	42.3		1.01 (0.79-1.29)	0.947
		CC	17/23	62.1		1.00 (0.60-1.67)	0.990	17/23	62.1		0.76 (0.46-1.26)	0.293
	Dominant	TT	133/182	58.1	0.950	Ref.		145/182	36.6	0.812	Ref.	
		CT/CC	121/166	60.4		0.99 (0.77-1.27)	0.922	131/166	45.6		0.97 (0.76-1.23)	0.788
	Recessive	CC	17/23	62.1	0.991	Ref.		17/23	62.1	0.238	Ref.	
		CT/TT	237/325	58.5		0.99 (0.61-1.62)	0.969	259/325	36.6		1.32 (0.80-2.15)	0.275
rs2249825	Additive	CC	193/264	58.4	0.599	Ref.		211/264	36.6	0.476	Ref.	
		GC	53/76	61.1		0.95 (0.70-1.29)	0.731	57/76	51.2		0.84 (0.62-1.13)	0.246
		GG	8/8	64.1		1.38 (0.68-2.80)	0.377	8/8	60.8		1.12 (0.55-2.27)	0.762
	Dominant	CC	193/264	58.4	0.820	Ref.		211/264	36.6	0.297	Ref.	
		GC/GG	61/84	61.3		0.99 (0.74-1.33)	0.942	65/84	54.1		0.87 (0.66-1.15)	0.317
	Recessive	GG	8/8	64.1	0.375	Ref.		8/8	60.8	0.750	Ref.	
		GC/CC	246/340	59.0		0.72 (0.35-1.46)	0.359	268/340	39.1		0.86 (0.43-1.75)	0.686
REV3L												
rs462779	Additive	CC	63/90	61.4	**0.018**	Ref.		68/90	52.6	0.106	Ref.	
		TC	127/185	60.1		0.94 (0.69-1.27)	0.671	141/185	36.6		1.01 (0.75-1.35)	0.949
		TT	64/73	50.3		1.42 (1.00-2.02)	**0.050**	67/73	27.8		1.30 (0.93-1.83)	0.131
	Dominant	CC	63/90	61.4	0.672	Ref.		68/90	52.6	0.481	Ref.	
		TC/TT	191/258	56.0		1.06 (0.79-1.41)	0.706	208/258	36.0		1.09 (0.83-1.43)	0.546
	Recessive	TT	64/73	50.3	**0.005**	Ref.		67/73	27.8	**0.034**	Ref.	
		TC/CC	190/275	60.5		0.67 (0.51-0.90)	**0.007**	209/275	42.3		0.77 (0.59-1.02)	0.073
rs465646	Additive	AA	164/219	60.0	0.423	Ref.		179/219	33.2	0.252	Ref.	
		GA	84/122	59.0		0.91 (0.69-1.18)	0.469	91/122	54.5		0.85 (0.66-1.10)	0.222
		GG	6/7	60.3		1.37 (0.60-3.12)	0.451	6/7	45.6		1.06 (0.47-2.41)	0.888
	Dominant	AA	164/219	60.0	0.393	Ref.		179/219	33.2	0.125	Ref.	
		GA/GG	90/129	59.1		0.93 (0.72-1.20)	0.572	97/129	54.0		0.86 (0.67-1.11)	0.250
	Recessive	GG	6/7	60.3	0.401	Ref.		6/7	45.6	0.719	Ref.	
		GA/AA	248/341	59.1		0.70 (0.31-1.60)	0.401	270/341	39.1		0.89 (0.40-2.02)	0.786
NFE2L2												
rs6706649	Additive	CC	224/304	59.1	0.900	Ref.		243/304	41.1	0.873	Ref.	
		CT	29/43	61.1		0.91 (0.62-1.35)	0.642	32/43	34.9		0.91 (0.63-1.33)	0.635
		TT	1/1	64.0		1.01 (0.14-7.29)	0.992	1/1	64.0		0.78 (0.11-5.64)	0.808
	Dominant	CC	224/304	64.0	0.860	Ref.		243/304	64.0	0.909	Ref.	
		CT/TT	30/44	59.1		0.98 (0.14-7.04)	0.981	33/44	39.1		1.26 (0.18-9.06)	0.820
	Recessive	TT	1/1	64.0	0.860	Ref.		1/1	64.0	0.909	Ref.	
		CT/CC	253/347	59.1		0.98 (0.14-7.04)	0.981	275/347	39.1		1.26 (0.18-9.06)	0.820
rs6721961	Additive	GG	126/176	59.8	0.547	Ref.		138/176	41.4	0.811	Ref.	
		TG	101/140	58.5		1.08 (0.83-1.41)	0.552	112/140	35.1		1.11 (0.87-1.43)	0.399
		TT	27/32	60.1		1.27 (0.84-1.94)	0.258	26/32	45.3		1.03 (0.68-1.57)	0.891
	Dominant	GG	126/176	59.8	0.466	Ref.		138/176	41.4	0.567	Ref.	
		TG/TT	128/172	58.5		1.12 (0.87-1.44)	0.378	138/172	35.8		1.10 (0.86-1.39)	0.444
	Recessive	TT	27/32	60.1	0.311	Ref.		26/32	45.3	0.926	Ref.	
		TG/GG	227/316	59.0		0.81 (0.55-1.22)	0.315	250/316	36.8		1.02 (0.68-1.53)	0.927
rs35652124	Additive	TT	71/99	60.5	0.951	Ref.		74/99	39.0	0.675	Ref.	
		CT	124/165	58.5		1.06 (0.79-1.43)	0.679	137/165	39.1		1.17 (0.88-1.55)	0.289
		CC	59/84	58.4		1.02 (0.72-1.44)	0.917	65/84	40.1		1.07 (0.77-1.50)	0.693
	Dominant	TT	71/99	60.5	0.799	Ref.		74/99	39.0	0.460	Ref.	
		CT/CC	183/249	58.5		1.05 (0.80-1.38)	0.735	202/249	39.1		1.13 (0.87-1.48)	0.360
	Recessive	CC	59/84	58.4	0.931	Ref.		65/84	40.1	0.848	Ref.	
		CT/TT	195/264	60.3		1.02 (0.76-1.37)	0.892	211/264	39.1		1.03 (0.78-1.36)	0.835

MST, median survival time; HR, hazard ratio; CI, confidence interval; P1, *P*-value for log-rank test; P2, *P*-value for multivariate Cox hazards regression with adjustment for age, gender, and histology. *P* ≤ 0.05 are indicated in bold text. Additive model: comparison between minor allele subjects and major allele subjects. Dominant model: comparison between minor allele carriers and major homozygous subjects. Recessive model: comparison between major allele carriers and minor homozygous subjects.

**Table 3:** Association between single nucleotide polymorphisms (SNPs) and lung cancer progression (n=305)

Gene/SNP	Genotype	T stage				N stage				M stage			
		T1/T2	T3/T4	OR (95% CI)	P	N0	N1/N2/N3	OR (95% CI)	P	M0	M1	OR (95% CI)	P
HMGB1													
rs1045411	GG	59	130	Ref.		39	150	Ref.		51	138	Ref.	
	AG/AA	57	59	0.47 (0.29-0.76)	**0.002**	16	100	1.63 (0.86-3.07)	0.134	37	79	0.79 (0.48-1.31)	0.358
rs1412125	TT	58	100	Ref.		35	123	Ref.		45	113	Ref.	
	CT/CC	58	89	0.89 (0.56-1.41)	0.622	20	127	1.81 (0.99-3.30)	0.054	43	104	0.96 (0.59-1.58)	0.882
	CC	6	11	Ref.		2	15	Ref.		3	14	Ref.	
	CT/TT	110	178	0.88 (0.32-2.45)	0.811	53	235	0.59 (0.13-2.66)	0.494	85	203	0.51 (0.14-1.83)	0.302
rs2249825	CC	79	150	Ref.		45	184	Ref.		65	164	Ref.	
	GC/GG	37	39	0.56 (0.33-0.94)	**0.028**	10	66	1.61 (0.77-3.39)	0.205	23	53	0.91 (0.52-1.61)	0.754
REV3L													
rs462779	CC	28	48	Ref.		7	69	Ref.		18	58	Ref.	
	TC/TT	88	141	0.94 (0.55-1.60)	0.805	48	181	0.38 (0.17-0.89)	**0.025**	70	159	0.71 (0.39-1.28)	0.252
	TT	30	38	Ref.		16	52	Ref.		20	48	Ref.	
	TC/CC	86	151	1.39 (0.80-2.40)	0.242	39	198	1.56 (0.81-3.01)	0.183	68	169	1.04 (0.57-1.87)	0.908
rs465646	AA	77	118	Ref.		34	161	Ref.		50	145	Ref.	
	GA/GG	39	71	1.19 (0.73-1.93)	0.486	21	89	0.90 (0.49-1.64)	0.718	38	72	0.65 (0.39-1.09)	0.100
NFE2L2													
rs6706649	CC	99	165	Ref.		46	218	Ref.		76	188	Ref.	
	CT/TT	17	24	0.85 (0.43-1.65)	0.627	9	32	0.75 (0.34-1.68)	0.484	12	29	0.98 (0.47-2.01)	0.950
rs6721961	GG	64	88	Ref.		33	119	Ref.		44	108	Ref.	
	TG/TT	52	101	1.41 (0.89-2.25)	0.145	22	131	1.65 (0.91-2.99)	0.098	44	109	1.01 (0.62-1.66)	0.971
	TT	13	18	Ref.		7	24	Ref.		8	23	Ref.	
	TG/GG	103	171	1.20 (0.56-2.55)	0.637	48	226	1.37 (0.56-3.37)	0.489	80	194	0.84 (0.36-1.97)	0.693
rs35652124	TT	38	52	Ref.		19	71	Ref.		24	66	Ref.	
	CT/CC	78	137	1.28 (0.78-2.12)	0.330	36	179	1.33 (0.72-2.47)	0.367	64	151	0.86 (0.49-1.49)	0.586
	CC	26	41	Ref.		12	55	Ref.		24	43	Ref.	
	CT/TT	90	148	1.04 (0.60-1.82)	0.883	43	195	0.99 (0.49-2.01)	0.976	64	174	1.52 (0.85-2.70)	0.156

OR, odds ratio; CI, confidence interval; P, P-value for binary logistic regression analysis; Ref., reference. P < 0.05 are indicated in bold text.
