# Supplementary material for: Association of variations in platinum resistance-related genes and prognosis in lung cancer patients
Source: J Cancer. 2020 Apr 27;11(15):4343–51. doi: 10.7150/jca.44410 (PMC7255368; doi:10.7150/jca.44410)
Supplement: Supplementary file 1 — Supplementary tables. [file jcav11p4343s1.pdf]

Supplementary Table 1. Stratified analysis of the association between rs462779 in REV3L gene and platinum-based chemotherapy prognosis (n=348)

Subgroups	Overall survival (OS)				Progression-free survival (PFS)			
	Death/Total		HR (95% CI)	P	Progression/total		HR (95% CI)	P
	TT	TC/CC			TT	TC/CC		
Age (years)								
≤56	36/42	87/129	0.61 (0.42-0.91)	**0.015**	38/42	101/129	0.83 (0.57-1.21)	0.328
>56	28/31	103/146	0.75 (0.49-1.15)	0.188	29/31	108/146	0.72 (0.47-1.10)	0.128
Gender								
Female	15/17	40/61	0.74 (0.39-1.41)	0.355	15/17	46/61	0.86 (0.47-1.58)	0.624
Male	49/56	150/214	0.69 (0.50-0.96)	**0.027**	52/56	163/214	0.77 (0.56-1.06)	0.115
Smoking status								
Never smoker	27/32	74/111	0.70 (0.44-1.09)	0.116	29/32	85/111	0.75 (0.49-1.16)	0.196
Ever smoker	37/41	116/164	0.65 (0.45-0.95)	**0.026**	38/41	124/164	0.80 (0.55-1.15)	0.229
Family history of cancer								
No	61/70	181/262	0.68 (0.51-0.92)	**0.011**	64/70	199/262	0.78 (0.59-1.04)	0.085
Yes	3/3	9/13	0.53 (0.09-3.15)	0.485	3/3	10/13	0.85 (0.17-4.12)	0.835
Histology								
LUAD	39/43	98/136	0.65 (0.45-0.94)	**0.023**	40/43	110/136	0.82 (0.57-1.18)	0.276
LUSC	20/24	78/118	0.80 (0.49-1.33)	0.395	21/24	83/118	0.85 (0.52-1.39)	0.517
Others[a]	5/6	14/21	1.14 (0.29-4.46)	0.848	6/6	16/21	0.56 (0.17-1.77)	0.320
TNM stage								
Ⅰ/Ⅱ	3/4	4/6	0.53 (0.10-2.81)	0.454	4/4	4/6	0.09 (0.01-1.00)	0.050
Ⅲ/Ⅳ	61/69	186/269	0.68 (0.51-0.91)	**0.009**	63/69	205/269	0.80 (0.60-1.07)	0.134
Eastern Cooperative Oncology Group Performance Status								
0-1	58/67	178/260	0.67 (0.49-0.90)	**0.008**	61/67	197/260	0.75 (0.56-1.01)	0.055
>1	6/6	12/15	0.48 (0.16-1.44)	0.192	6/6	12/15	1.36 (0.41-4.58)	0.617

HR, hazard ratio; CI, confidence interval; P, *P*-value for multivariate Cox hazards regression analysis with adjustment for age, gender, and histology; Ref.,reference; LUAD, lung adenocarcinoma; LUSC, lung squamous cell carcinoma. $P < 0.05$ are indicated in bold text.

[a]Other carcinomas include adenosquamous carcinoma, large cell carcinoma, bronchogenic lung cancer, and mucoepidermoid carcinoma.

Supplementary Table 2. Stratified analysis of the association between rs1045411 in HMGB1 gene and platinum-based chemotherapy prognosis (n=348)

Subgroups	Overall survival (OS)				Progression-free survival (PFS)			
	Death/Total		HR (95% CI)	P	Progression/total		HR (95% CI)	P
	AA	AG/GG			AA	AG/GG		
Age (years)								
≤56	10/10	113/161	0.54 (0.28-1.04)	0.064	10/10	129/161	0.82 (0.43-1.56)	0.540
>56	8/8	123/169	0.68 (0.33-1.41)	0.300	8/8	129/169	0.84 (0.41-1.73)	0.639
Gender								
Female	6/6	49/72	0.58 (0.24-1.37)	0.214	6/6	55/72	0.90 (0.38-2.13)	0.818
Male	12/12	187/258	0.64 (0.35-1.15)	0.135	12/12	203/258	0.84 (0.47-1.52)	0.572
Smoking status								
Never smoker	8/8	93/135	0.50 (0.24-1.04)	0.062	8/8	106/135	0.82 (0.40-1.69)	0.588
Ever smoker	10/10	143/195	0.75 (0.37-1.52)	0.422	10/10	152/195	0.91 (0.45-1.81)	0.779
Family history of cancer								
No	17/17	225/315	0.63 (0.39-1.04)	0.071	17/17	246/315	0.85 (0.52-1.39)	0.516
Yes	1/1	11/15	0.15 (0.01-1.93)	0.145	1/1	12/15	0.33 (0.03-3.42)	0.354
Histology								
LUAD	11/11	126/168	0.63 (0.34-1.18)	0.147	11/11	139/168	0.96 (0.52-1.78)	0.896
LUSC	6/6	92/136	0.69 (0.30-1.59)	0.381	6/6	98/136	0.73 (0.32-1.69)	0.462
Others[a]	1/1	18/26	ns	1.000	1/1	21/26	0.25 (0.02-2.90)	0.265
TNM stage								
Ⅰ/Ⅱ	0/0	7/10	NA	NA	0/0	8/10	NA	NA
Ⅲ/Ⅳ	18/18	229/320	0.62 (0.38-1.00)	**0.050**	18/18	250/320	0.84 (0.52-1.36)	0.484
Eastern Cooperative Oncology Group Performance Status								
0-1	17/17	219/310	0.57 (0.35-0.93)	**0.025**	17/17	241/310	0.77 (0.47-1.27)	0.309
>1	1/1	17/20	0.63 (0.05-7.61)	0.713	1/1	17/20	0.47 (0.04-6.00)	0.559

HR, hazard ratio; CI, confidence interval; P, *P*-value for multivariate Cox hazards regression analysis with adjustment for age, gender, and histology; Ref., reference; LUAD, lung adenocarcinoma; LUSC, lung squamous cell carcinoma. $P \leq 0.05$ are indicated in bold text.

[a]Other carcinomas include adenosquamous carcinoma, large cell carcinoma, bronchogenic lung cancer, and mucoepidermoid carcinoma.
